# Proline-mediated redox regulation in wheat for mitigating nickel-induced stress and soil decontamination

**DOI:** 10.1038/s41598-023-50576-5

**Published:** 2024-01-03

**Authors:** Nimra Atta, Muhammad Shahbaz, Fozia Farhat, Muhammad Faisal Maqsood, Usman Zulfiqar, Nargis Naz, Muhammad Mahmood Ahmed, Naveed Ul Hassan, Nazoora Mujahid, Abd El-Zaher M. A. Mustafa, Mohamed S. Elshikh, Talha Chaudhary

**Affiliations:** 1 Department of Botany, University of Agriculture, Faisalabad, Pakistan; 2 Department of Botany, Government College Women University, Faisalabad, Pakistan; 3 Department of Botany, The Islamia University of Bahawalpur, Bahawalpur, 63100 Pakistan; 4 Department of Agronomy, Faculty of Agriculture and Environment, The Islamia University of Bahawalpur, Bahawalpur, 63100 Pakistan; 5 Department of Bioinformatics, The Islamia University of Bahawalpur, Bahawalpur, 63100 Pakistan; 6 Department of Botany and Microbiology, College of Science, King Saud University, P.O. 2455, Riyadh, 11451 Saudi Arabia; 7 Faculty of Agricultural and Environmental Sciences, Hungarian University of Agriculture and Life Sciences, Godollo, 2100 Hungary

**Keywords:** Physiology, Plant sciences, Natural variation in plants, Plant hormones

## Abstract

Nickel (Ni) is known as a plant micronutrient and serves as a component of many significant enzymes, however, it can be extremely toxic to plants when present in excess concentration. Scientists are looking for natural compounds that can influence the development processes of plants. Therefore, it was decided to use proline as a protective agent against Ni toxicity. Proline (Pro) is a popularly known osmoprotectant to regulate the biomass and developmental processes of plants under a variety of environmental stresses, but its role in the modulation of Ni-induced toxicity in wheat is very little explored. This investigation indicated the role of exogenously applied proline (10 mM) on two wheat varieties (V1 = Punjab-11, V2 = Ghazi-11) exposed to Ni (100 mg/kg) stress. Proline mediated a positive rejoinder on morphological, photosynthetic indices, antioxidant enzymes, oxidative stress markers, ion uptake were analyzed with and without Ni stress. Proline alone and in combination with Ni improved the growth, photosynthetic performance, and antioxidant capacity of wheat plants. However, Ni application alone exhibited strong oxidative damage through increased H2O2 (V1 = 28.96, V2 = 55.20) accumulation, lipid peroxidation (V1 = 26.09, V2 = 38.26%), and reduced translocation of macronutrients from root to shoot. Application of Pro to Ni-stressed wheat plants enhanced actions of catalase (CAT), peroxidase (POD), superoxide dismutase (SOD), and total soluble protein (TSP) contents by 45.70, 44.06, 43.40, and 25.11% in V1, and 39.32, 46.46, 42.22, 55.29% in V2, compared to control plants. The upregulation of antioxidant enzymes, proline accumulation, and uptake of essential mineral ions has maintained the equilibrium of Ni in both wheat cultivars, indicating Ni detoxification. This trial insight into an awareness that foliar application of proline can be utilized as a potent biochemical method in mitigating Ni-induced stress and might serve as a strong remedial technique for the decontamination of polluted soil particularly with metals.

## Introduction

Heavy metal pollution, either prevailing naturally or anthropogenically, poses a significant threat to sustainable agricultural productivity^1–4^. They are extremely persistent in soil owing to their non-biodegradability^5–7^, which results in stunted plant growth, chlorosis, root browning, and death of the entire plan^8,9^. Heavy metals are usually grouped as according to their physiological significance to the plant metabolic activities^10^. Nickel (Ni) is considered as micronutrient ranging between 0.01 and 5 µg g^−1^ dry weight of the plants^11^. Nickel is involved in the regulation of many enzymes including glyoxalase-I system urease, superoxide dismutases (SOD), and hydrogenase^12^. However, excessive amounts of Ni can lead to plant toxicity. Environmental pollution from Ni may be due to industrialization, the use of liquid and solid fuels, municipal and industrial waste and the injudicious use of inorganic fertilizers^13^. The contamination of Ni has been increasing worldwide in recent years, including Asia, North America and Europe^14^. Studies have recorded concentrations of Ni in aquatic environments reaching levels of up to 0.2 mg/L and in terrestrial resources up to 26 g/kg. This amount is approximately 25 times higher than in resources not affected by Ni contamination^15^. When Ni concentrations exceed the recommended range, it negatively impacts plant physiology. High Ni levels have been shown to reduce plant weight, hinder photosynthetic activity and respiration, impede mineral intake, and disrupt the nitrogen metabolic pathway^16^. Additionally, elevated Ni levels can induce the release of harmful reactive oxidant species (ROS) that can disorder the antioxidant immune system, leading to oxidative damage in plants^17^. One of the significant consequences of Ni toxicity is the reduced absorption of essential minerals and nutrients like iron, copper, manganese, and zinc by plants, which can result in nutrient deficiencies^18^. Plants alleviate heavy metal induced oxidative damage by releasing defensive regulatory molecules like antioxidants and osmoprotectants^19–22^.

Wheat (*Triticum aestivum* L.) is popularly cultivated crop among the cereals, serving as a staple food globally^23^. With a current yearly production of 786.49 million metric tons of wheat, fulfill the demand of the growing population around the word but the requirement of wheat production is predicted to increase by 2050 up to 70% increase^24^. Wheat bran and wheat germ offer unique ingredients for food applications, such as dietary fiber, minerals, B vitamins, lipids, and protein^25^. Its rich nutritional content, with 60–80% carbohydrates and 8–15% protein, makes wheat superior to other grains^26^. Wheat grain yield and quality are affected by genotype, environmental conditions, and interactions between the two^27,28^. Nickel is considered as readily stored element in the wheat grains even at a concentration as low as 200 mg kg^−1^, shows serious health concerns. Excess Ni in wheat seedlings may prevent the translocation of other essential micronutrient like Cu, and Mg. Increased Ni concentration can disrupt the absorption and distribution of mineral elements in different plant organs, causing Ni toxicity in wheat^29^.

Proline, known as low molecular compounds boosts plant tolerance against variety of heavy metals stress and other abiotic stresses^30,31^. These molecules salvage plants and create osmotic adjustment, thus improving stress tolerance^32^. Proline, acting as a solute, osmoregulator, and amino acid, plays a defensive role by detoxifying reactive oxygen species, protecting membranes, and stabilizing enzymes and proteins^33^. It maintains membrane integrity, prevents electrolyte seepage, and reduces reactive oxygen species levels^34,35^. Proline application has been shown to enhance stress tolerance in various food crops, including cucumber, pea, rice, and sunflower, when applied at appropriate concentrations^36,37^. Under stress conditions, proline accumulates in plants, aiding in osmoregulation and protecting cell membranes and organs. It serves as a primary solute, shielding plants from oxidative damage and stress, and also safeguards chloroplasts, mitochondria, and DNA. Additionally, it promotes photosynthesis and sugar accumulation in plants^38^. Proline application in stressed plants induces structural changes, increasing root surface area to overcome water and nutrient deficiencies. Moreover, it stimulates root growth and induces structural changes in leaves and stems of stressed plants^39^.

Proline plays a diverse role in plants, especially under harsh environmental conditions. It performs a crucial role in regulating the Ni tolerance in sensitive crops. However, the protective role of proline in alleviating Ni phytotoxicity is not much explored. So, this study aimed to explore the effect of proline on the regulation of morphological, physiological and biochemical parameters of wheat under Ni stress. Enhancing tolerance through proline may expand wheat cultivation into Ni-contaminated soil, contributing to global food security by maximizing the growth. The results of this study are expected to deepen our understanding regarding proline-mediated regulatory mechanism of wheat tolerance to Ni stress.

## Methodology

**Figure 1:**
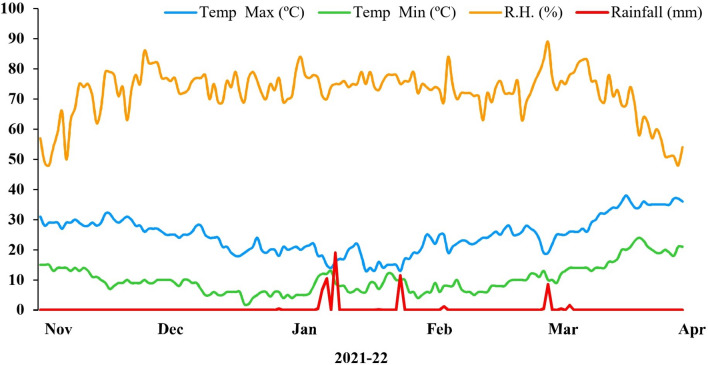
Weather date during the growing season 2021–22.

The experiment was executed at the experimental area, University of Agriculture, Faisalabad (Latitude: 32° 26´ 45.7046", Longitude: 74° 5´ 13.2811") to examine the influence of proline as foliar treatment on wheat under Ni stress. The experiment with completely randomized design had 4 replicates per treatment. The two wheat varieties were exploited namely Punjab-11 (VI) and Ghazi-11 (V2). Treatments for the experiment were T0 (0 mM Proline + 0 µM Ni), T1 (10 mM Proline + 0 µM Ni), T2 (0 mM Proline + 100 mg/kg Ni) and T3 (10 mM Proline + 100 mg/kg Ni). For sowing, each pot (28 cm × 24 cm × 21.5 cm) carrying 8 kg of soil was provided with twelve seeds. Plants were watered twice every week. After two weeks of seed sowing, thinning was done for maintaining 6 plants per pot. Nickel stress (100 mg kg^−1^) was imposed at early boot stage (BBCH identification code 41^40^ and foliar application of proline was given as 0 and 10 mM concentration at end of heading stage (BCCH identification code 59)^40^. Weather data for crop duration November 2021 to April 2022 is presented in Fig. 1. Plants were harvested after 3 weeks of foliar application to record different parameters.

## Plant sampling

Four healthy plants were uprooted from each pot. The two plants were used for recording morphological parameters (fresh and dry weights and length of root and shoot) and the leaves of other two plants were stored at − 20 °C to determine physiological and biochemical attributes.

### Plant analyses

#### Morphological parameters

##### Length and weight

The length of shoot and root were recorded in “cm” using measurement tape while fresh and dry weights were recorded in “g” using weighing balance.

#### Biochemical parameters

##### Photosynthetic pigments

Protocol devised by Arnon^41^ was followed for determining the pigments involved in photosynthesis. Fresh leaves (0.1 g) were extracted in 80% acetone. The solution was kept overnight in the dark. The ultraviolet visible spectrophotometer (U2020 IRMECO) was utilized for taking the readings at 480, 645 and 663 nm.

##### Enzymatic antioxidants

Flag leaves of two wheat cultivars (0.25 g) were extracted in 5 mL of potassium phosphate buffer (pH 7.8) at 4 °C and centrifuged at 12,000 rpm for 15 min. Activities of enzymatic antioxidants were determined exploiting supernatant.

##### Catalase (CAT)

The technique developed by Chance and Maehly^42^ was tracked for investigating the CAT concentration in both stressed and non-stressed wheat cultivars. Each cuvette had phosphate buffer (1.9 mL), H_2_O_2_ (1 mL) and plant extract (0.1 mL). Absorbance was noted for 2 min after every 30 s at 240 nm.

##### Peroxidase (POD)

In accordance with the process developed by Chance and Maehly^42^, the accumulation pattern of POD was estimated. Each sample contains a measured and standard volume of phosphate buffer, guaiacol (20 mM), H_2_O_2_ (40 mM) and 50 µL plant extract. The readings were taken at 470 nm after every 30 s for 2 min.

##### Superoxide dismutase (SOD)

The assessment of the activity of SOD activity was done by following Method by Spitz and Oberley^43^. The reaction mixture consisting of distilled water, phosphate buffer, L-Methionine, Triton-X, NBT, riboflavin and plant sample (50 µL), in a total volume of 2.5 mL was placed underneath a fluorescent lamp for 15–20 min. The readings were noted at 560 nm with a regular interval of 30 s interval for 2 min.

##### Total soluble proteins

The assessment of total soluble proteins was done by pursuing the protocol of Bradford^44^. The previously extracted antioxidant solution was used for estimating total soluble proteins (TSP). The Bradford reagent (5 mL) was mixed with enzyme extract and vortexed for 5 s. Absorbance was noted at 595 nm with UV-spectrophotometer.

##### Reactive oxygen species (ROS)

##### 1Hydrogen peroxide (H_2_O_2_)

H_2_O_2_ determination followed Velikova et al.^45^. Frozen leaf tissue was extracted in 0.1% TCA, centrifuged at 12,000 rpm for 20 min. Equal volumes of supernatant, pH 7.00 potassium phosphate buffer, and 1 mL KI were mixed in a glass tube. Absorbance was measured at 390 nm after vortexing.

##### Lipid peroxidation markers

##### Malondialdehyde (MDA)

The concentration of MDA was estimated by following Cakmak and Horst^46^. The frozen leaves were ground in 0.1% TCA, followed by centrifugation at 13,000 rpm for 15 min. The upper transparent liquid was further diluted with 4 mL solution containing 20% thiobarbituric acid (TBA) fortified with 0.5% TCA, and boiled at 100 °C for 30 min. Reading for each sample was taken at 532 nm and 600 nm after being cooled in ice.

##### Anthocyanins

For the estimation of anthocyanins, Stark and Wray’s^47^ bioassay was employed. Leaf samples of each treatment and replicate was separately chopped in small pieces, supplemented with 2 mL of acidified methanol and boiled at 90 °C for 1 h and optical density was noted at 535 nm.

##### Total soluble sugars

Approximation of total soluble sugars was performed out by adopting protocol designed by Yoshida et al.^48^. The leaf sample (0.1 g) was treated with deionized water (10 mL) and heated at 90 °C for 1 h. Dilution was done with deionized water to get desired volume. The anthrone reagent (5 mL) was mixed with this dilution (1.5 mL) and again heated 90 °C for 20 min. Absorbance was noted at 620 nm.

##### Flavonoids

The method developed by Zhishen et al*.*^49^ for the evaluation of flavonoids was followed. The 0.1 g leaf sample was mixed with 80% acetone and kept overnight in dark. The 1 mL of this solution was supplemented with distilled water, 5% NaNO_2_, 10% AlCl_3_, and 1 M NaOH. Optical density was noted down at 510 nm.

##### Ascorbic acid (AsA)

The AsA contents were recorded through subsequent procedure of Mukherjee and Choudhuri^50^. Leaf sample was ground in 6% TCA solution and centrifuged at 12,000 rpm for 10 min. The 2% dinitrophenyl hydrazine (1 mL) along with a tiny drop of 10% thiourea were added to the supernatant, subjected to heating in boiling water container at 90 °C for 15 min, later cooled down in ice. The freshly prepared sulphuric acid (80%) was added, and reading was taped at 530 nm.

### Mineral nutrients

#### Na^+^, K^+^ and Ca^2+^

Dried shoot sample (0.1 g) was digested with 3 mL sulphuric acid and kept overnight to be digested at maximum. The reaction mixture for each sample was placed on hot plate and hydrogen peroxide (5 mL) was added. Hydrogen peroxide was added again until the solution became colorless. The solution was then allowed to cool after removing from hot plate and filtered. Dilution was done by adding distilled water until the final volume became 50 mL. The reading was taken on flame photometer (Sherwood-410) for the determining Na^+^, K^+^ and Ca^2+^.

### Statistical analysis

Three-way analysis of variance (ANOVA) was implicated and data significance was analysed by using Statistix 8.1 at significance level of 0.05. Principal component analysis (PCA), heat plot and correlation analysis were performed through R-studio (version R-4.3.1).

### Plant guidelines

All the plant experiments were performed by relevant institutional, national, and international guidelines and legislations.

## Results

### Proline reduced inhibitory effects of Ni by enhancing biomass production of wheat seedlings

**Figure 2:**
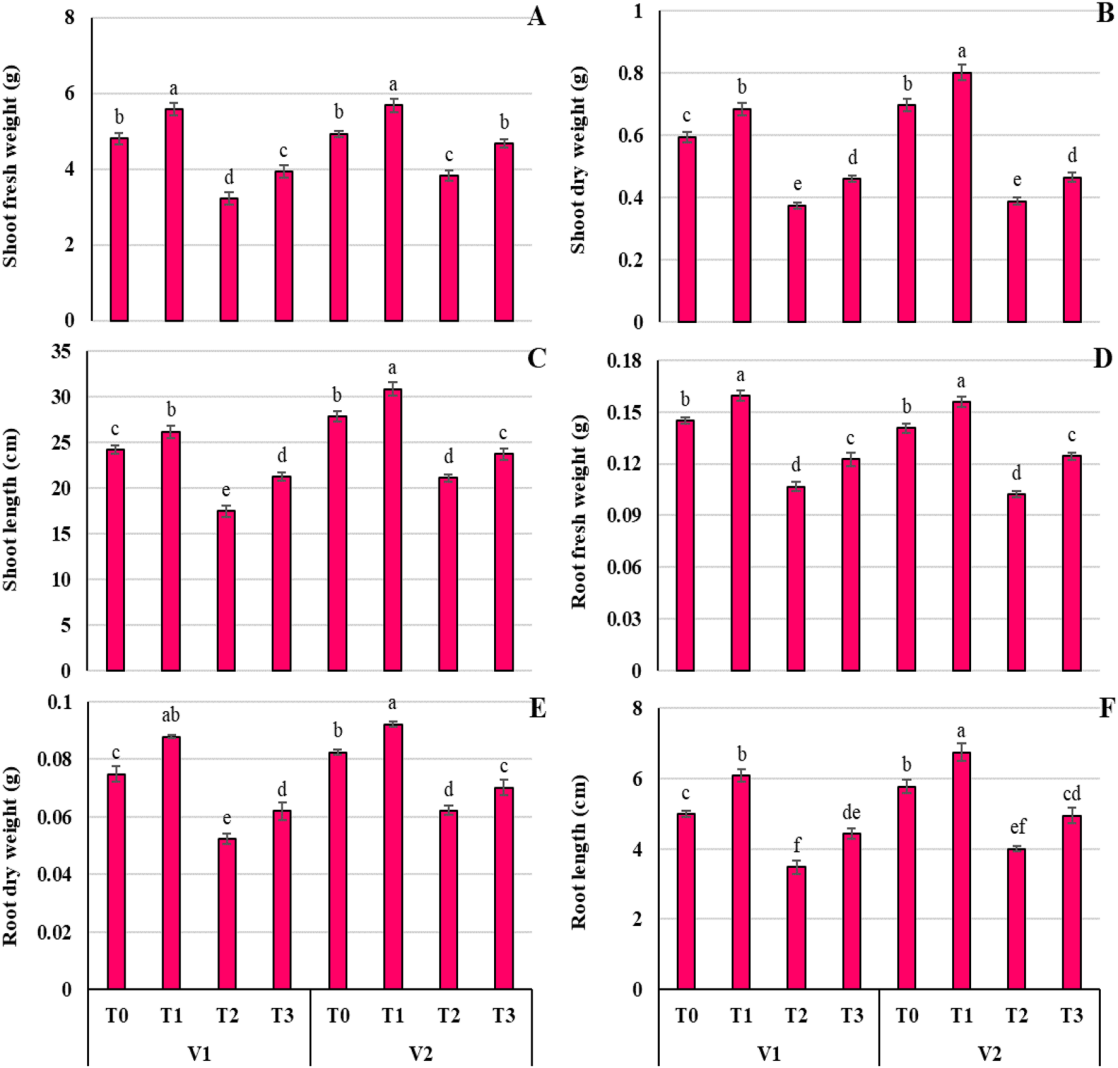
Effect of Ni (100 mg/kg) stress with and without foliar application of Pro (10 mM) on shoot fresh weight (**A**), shoot dry weight (**B**), shoot length (**C**), root fresh weight (**D**), root dry weight (**E**), and root length (**F**) in two varieties (V1 = Punjab-11, V2 = Ghazni-11) of *Triticum aestivum* L. seedlings.

Figure 2A–F describes some of the growth features of the two wheat varieties (V1 = Punjab-11, V2 = Ghazi-11), such as shoot fresh and dry weight (SF/DW), shoot length (SL), root fresh and dry weight (RF/DW) and root length (RL) when treated with Ni (100 mg kg^−1^) and proline (10 mM), along with no treatment (T_0_) and proline (Pro) application alone (T_1_). The application of Ni to both wheat varieties drops the growth parameters (Fig. 2).The shoot length of both varieties V1 and V2 was substantially (*P* ≤ 0.001) lowered when the plants were watered containing 100 mg kg^−1^ of Ni, by ^~^27.83% and 24.28%, respectively, contrary to the plants without stress of their respective wheat variety. Similarly, Ni treatment caused a drastic (*P* ≤ 0.001) reduction of SFW (26.53%), RFW (26.92%), RDW (27.58%), and RL (30.53%) in both varieties. On the contrary, foliar application of proline (T_3_) significantly (*P* ≤ 0.05) reduced the inhibitory effect of Ni, by limiting the reduction of SFW (1A), SDW (1B), SL(1C), RFW (1D), RDW (1E), and RL (1F) up to 11.59, 24.38, 13.52, 13.50, 16.02, and 12.65%, respectively compared to T_0_ treatment of both varieties. Both wheat varieties showed significant differences, as V1 showed a more drastic reduction of growth characteristics compared to V2, but the recovery mechanism was more pronounced in V1 with Pro treatment facing Ni toxicity (T_3_).

### Proline alleviates the photosynthesis reduction induced by Ni stress in wheat seedlings

**Figure 3:**
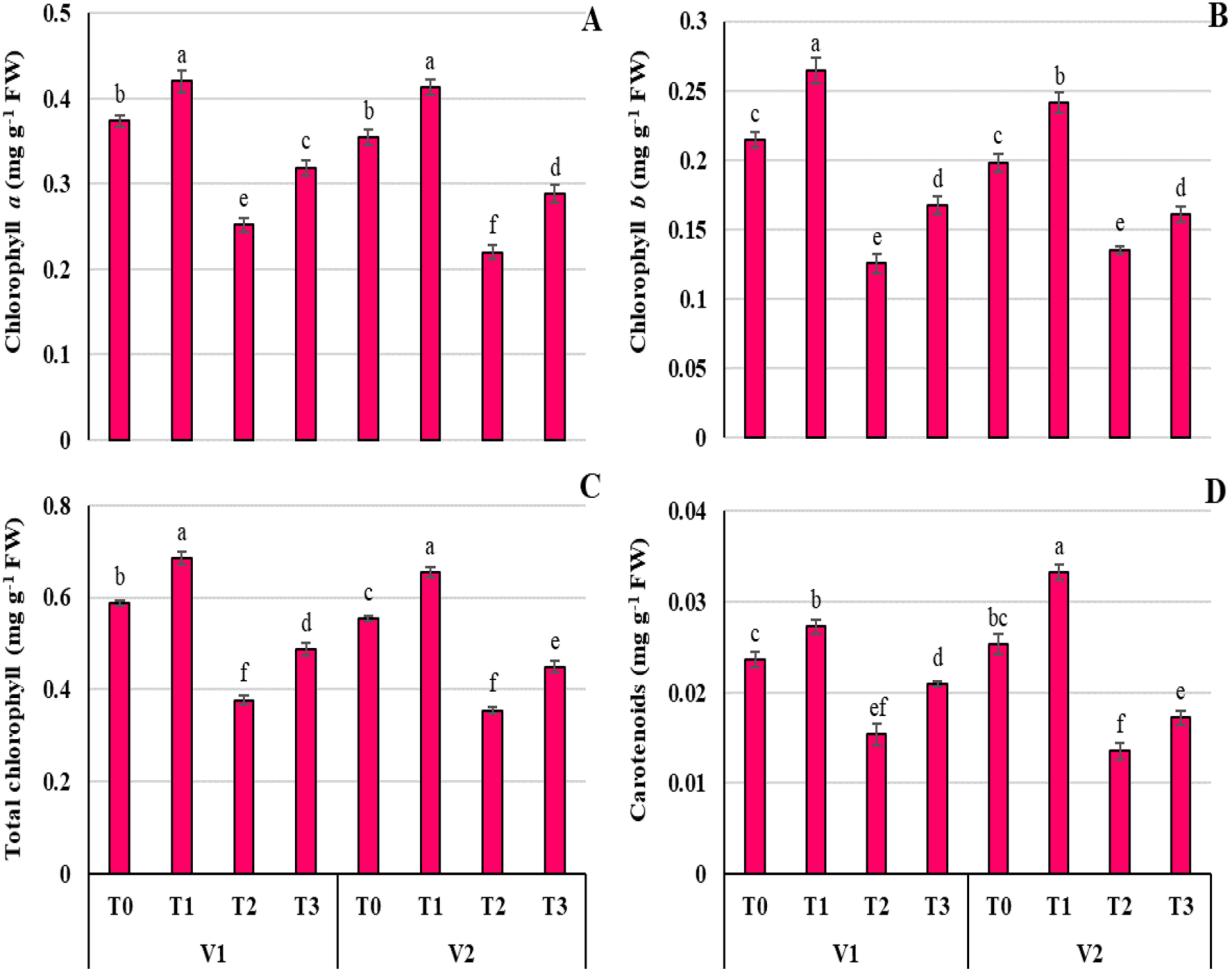
Effect of Ni (100 mg/kg) stress with and without foliar application of Pro (10 mM) on chlorophyll *a* (**A**), chlorophyll *b* (**B**), total chlorophyll (**C**), carotenoid (**D**) contents in two varieties (V1 = Punjab-11, V2 = Ghazni-11) of *Triticum aestivum* L. seedlings.

In plants treated to Ni stress, chlorophyll *a*, chlorophyll *b*, total chlorophyll and carotenoids levels fell by 36.97, 36.75, 35.78, and 40.65%, respectively, in relevance to control plants (Fig. 3A-D). In comparison to Ni-stressed plants, Pro treatment increased (*P* ≤ 0.01) the chlorophyll *a*, chlorophyll *b*, total chlorophyll, and carotenoid contents by 29.64%, 18.39%, 37.07%, and 32.55%. Proline also enhanced the amounts of chl. *a*, chl. *b*, total chl*.* and carotenoids in non-stressed plants by 14.46, 22.48, 17.38, and 23.33%, respectively (Fig. 3). All photosynthetic pigments showed a non-significant difference in both wheat varieties except carotenoids, where V2 showed higher accumulation of carotenoids with Pro treatment with (T_3_) and without stress (T_1_).

### Proline elevates the endogenous antioxidant pooling Ni-stressed wheat seedlings

**Figure 4:**
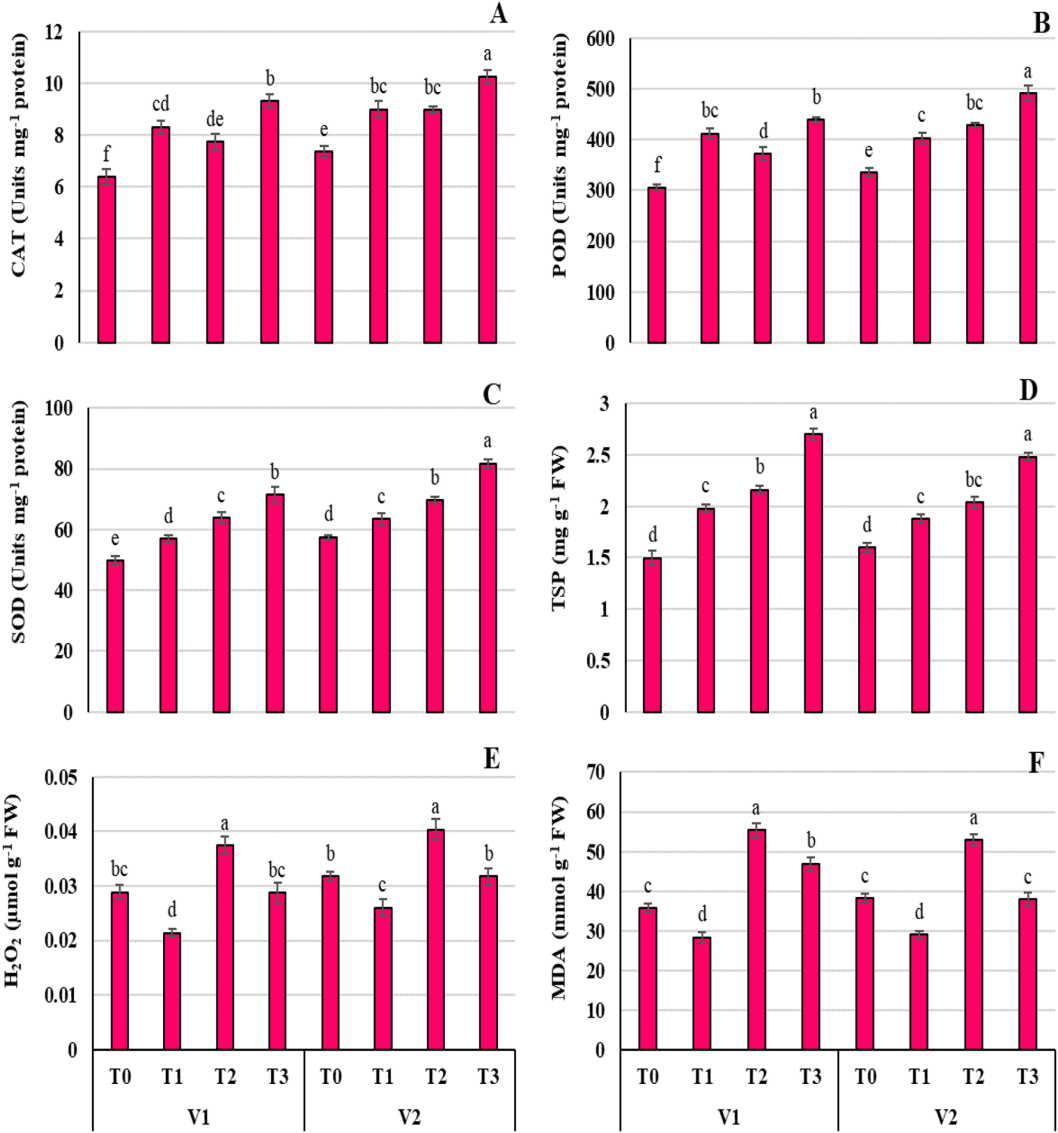
Effect of Ni (100 mg/kg) stress with and without foliar application of Pro (10 mM) on catalase (**A**), peroxidase (**B**), superoxide dismutase (**C**), total soluble protein (**D**), hydrogen peroxide (**E**), and malondialdehyde (**F**) contents in two varieties (V1 = Punjab-11, V2 = Ghazni-11) of *Triticum aestivum* L*.* seedlings.

To verify the antioxidant role of exogenous 10 mM Pro under Ni stress, the contents of CAT, POD, SOD and TSP were recorded in wheat plants. Under only Ni treatment (T_2_), a significant (*P* ≤ 0.05) increase was observed in CAT (V1 = 21.09, V2 = 21.69%), POD (V1 = 21.86, V2 = 27.54%), SOD (V1 = 28.11, V2 = 21.21%), and TSP (V1 = 1.68 V2 = 27.62%) contents compared to non-stressed plants (Fig. 4). Furthermore, CAT, POD, SOD, TSP contents were also significantly (*P* ≤ 0.001) increased by 45.70, 44.06, 43.40, and 25.11% in V1, and 39.32, 46.46, 42.22, 55.29% in V2 under Pro + Ni treatment relative to control plants (4A-D).

### Proline alleviates the endogenous oxidative damage induced in Ni-stressed wheat seedlings

To foster the impact of Pro under Ni, reactive oxygen species (ROS) in non-radical form H_2_O_2_ (Fig. 4E) and MDA (Fig. 4F) content were examined. The important findings specified that Ni toxicity has pointedly (*P* ≤ 0.001) increased endogenous H_2_O_2_, and MDA concentration by 28.96, 55.20% in V1, and 26.09, 38.26% in V2 wheat seedlings, respectively. However, oxidative damage was minimized (*P* ≤ 0.05) by the exogenous treatment of Pro (Fig. 4). In comparison to Ni treatment alone, Ni + Pro application appeared to reduce H_2_O_2_ and MDA contents by 9.32–22.43%, and 15.25–28.42% in both wheat varieties, respectively (Fig. 4E,F).

### Proline elevates osmolyte accumulation in Ni-stressed Wheat seedlings

**Figure 5:**
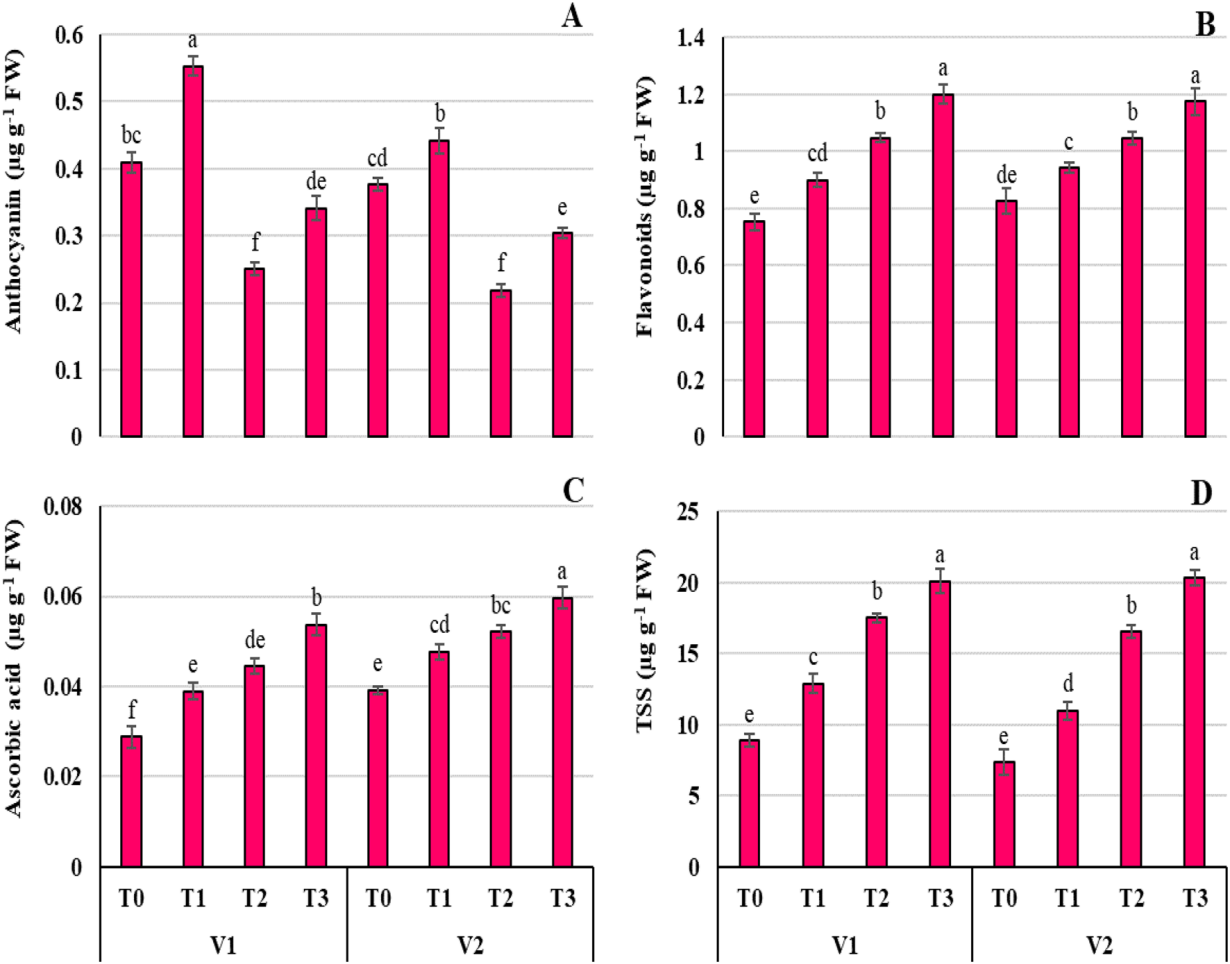
Effect of Ni (100 mg/kg) stress with and without foliar application of Pro (10 mM) on anthocyanin (**A**), flavonoid (**B**), ascorbic acid (**C**), and total soluble sugar (**D**) contents in two varieties (V1 = Punjab-11, V2 = Ghazni-11) of *Triticum aestivum* L*.* seedlings.

The exposure of wheat plants to Ni stress leads to more accumulation of essential organic osmolytes which have a significant key role in regulating growth under stress. Proline-treated plants in combination with Ni (T_3_) showed a significant (*P* ≤ 0.05) increase of anthocyanin (Fig. 5A), flavonoid (Fig. 5B), ascorbic acid (AsA; Fig. 5C), and total soluble sugars (TSS; Fig. 5D) contents in both wheat varieties. Nickel stress significantly (*P* ≤ 0.001) reduced anthocyanin contents in V1 (38.90%) and V2 (41.73%). However, the combined application of Ni + Pro significantly (*P* ≤ 0.05) enhanced anthocyanin activity in both wheat cultivars (V1 = 16.72%, V2 = 19.32%). Under Ni stress alone (T_2_) and Pro and Ni stress (T_3_), flavonoid contents increased by 32.78% and 50.59%, respectively (Fig. 5B). Similarly, AsA content was enriched in Pro treated plants alone and in Ni + Pro plants. Introduction of Ni in the rhizosphere to growing plants increased AsA content by 54.48% while Pro + Ni treated plants showed an increase of approximately 86.71% over the control plants of V1 (Fig. 5C). Under Ni stress alone (T_2_) and Pro + Ni (T_3_) treatment, TSS activity increased by 96.26–125.11% and 125.73–176.60% respectively, in both wheat varieties (Fig. 5D).

### Pro application elevates the Ca^2+^ and K^+^ while suppressed Na^+^ in Ni-stressed Wheat seedlings

**Figure 6:**
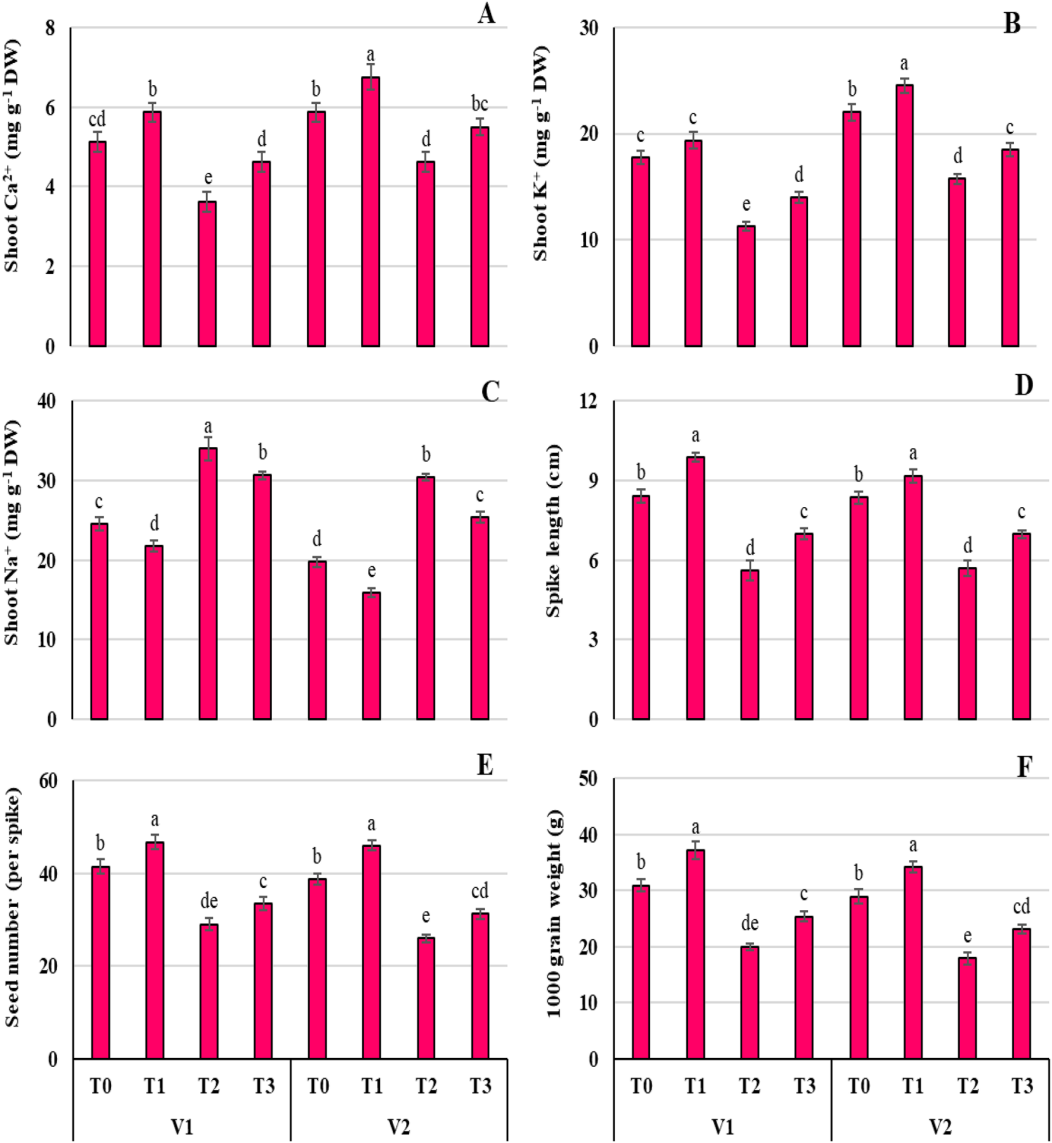
Effect of Ni (100 mg/kg) stress with and without foliar application of Pro (10 mM) on shoot potassium ions (**A**), shoot calcium ions (**B**), shoot sodium ions (**C**), spike length (**D**), seed number per spike (**E**), and 1000 grain weight (**F**) in two varieties (V1 = Punjab-11, V2 = Ghazni-11) of *Triticum aestivum* L. seedlings.

Nickel has caused osmotic disparity in both wheat varieties (Fig. 6). The endogenous potassium ions (K^+^), and calcium ions (Ca^2+^) concentration drops substantially (*P* ≤ 0.05) in both wheat seedlings under Ni conditions (T_2_), related to their respective control plants (T_0_) (Fig. 6A-C). The foliar application of Pro (T_3_) reduced the inhibitory effect of Ni on Ca^2+^ and K^+^ accumulation by 6.38, and 15.91% compared to drastic reduction under T_2_ (21.28, 28.41%) in V1, while T_1_ treatment significantly (*P* ≤ 0.01) enhanced Ca^2+^ (Fig. 5A; 14.63, 14.89%), and K^+^ (Fig. 6B; 9.15, 11.36%) in both wheat varieties.

Nickle stress caused a significant (*P* ≤ 0.001) higher accumulation of toxic ions like Na^+^ in shoot samples of both wheat varieties (Fig. 6C). The Na^+^ accumulated up to38.78 and 53.83% under Ni stress (T_2_) in both wheat varieties, when compared with respective control wheat variety. However, Pro + Ni treatment (T_3_) substantially (*P* ≤ 0.01) minimized Na^+^ accumulation in V1 (25.00%) and V2 (28.45%). The ionic fluxes after recovery from Ni with Pro treatment were considerably altered in both wheat varieties (Fig. 6A-C).

### Proline alleviates the yield reduction induced by Ni stress in wheat seedlings

The Ni stress condition considerably (*P* ≤ 0.01) reduced the overall yield of both wheat cultivars (Fig. 6D-F). When both varieties were subjected to Pro application under non-stress condition (T_1_), spike length (6D), number of seeds per spike (Fig. 6E), and 1000 grain weight (Fig. 6F), increased up to 16.92, 19.23, and 19.36% respectively. However, Ni-affected plants (T_2_) of both varieties exhibited reduced spike length (V1 = 33.53, V2 = 32.03%), number of seeds per spike (V1 = 30.12, V2 = 32.90%), and 1000 grain weight (V1 = 35.25, V2 = 38.20%), compared to non-stressed plants (T_0_). The foliar-applied treatment (T_3_) minimized the inhibitory effect of Ni stress compared to their stressed plants (T_2_). Overall, both cultivars showed non-significant differences in their agronomic attributes (Fig. 6D-F).

### Principal Component Analysis (PCA), Heat-plot and correlation analysis:

**Figure 7:**
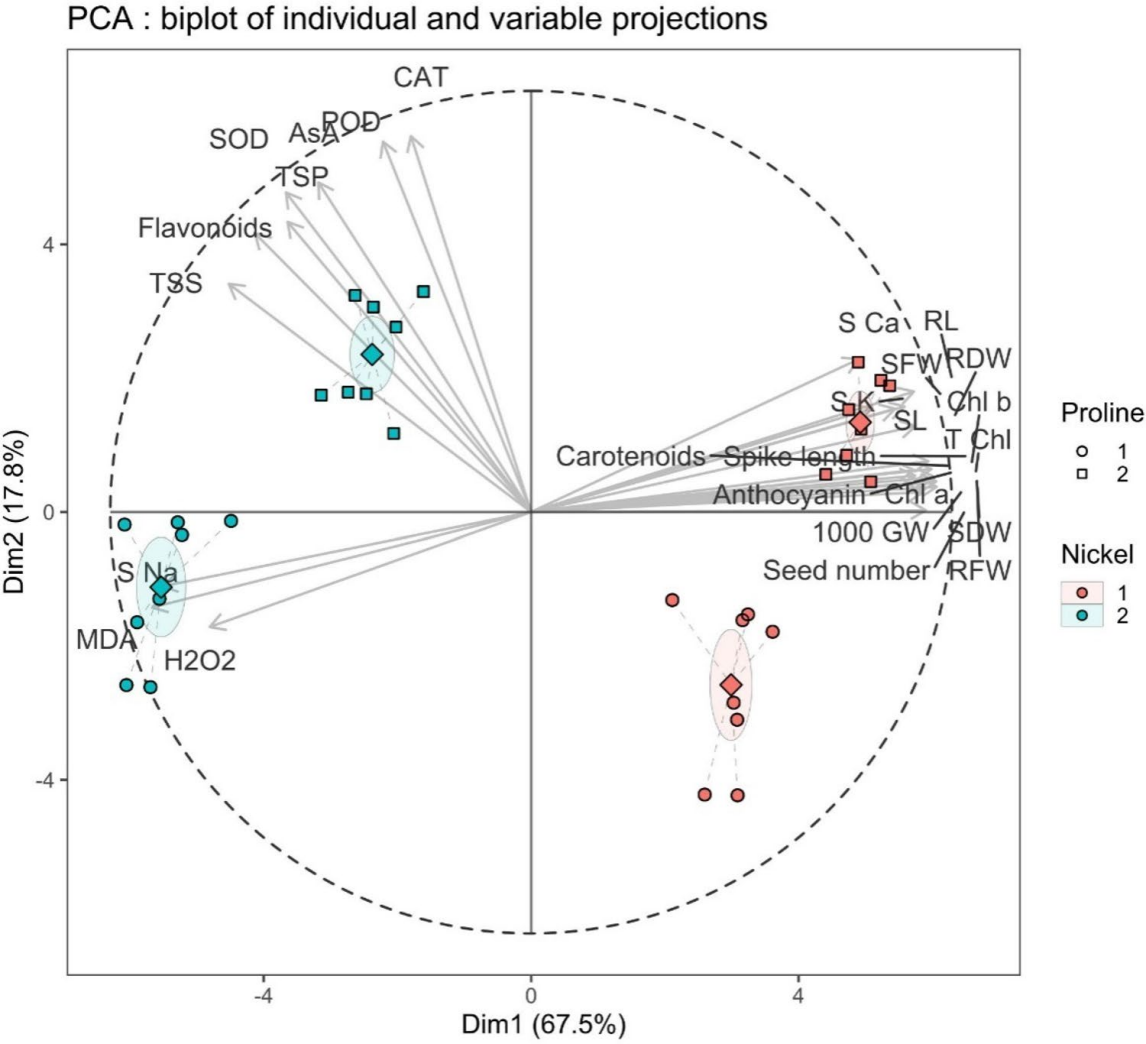
PCA biplot of both individual and variable projections under Proline and Nickel treatments. (CAT: catalase, POD: peroxidase, AsA: Ascorbic acid, SOD: superoxide dismutase, TSP: total soluble proteins, TSS: total soluble sugars, S Na: shoot sodium, MDA: malondialdehyde, H_2_O_2_: hydrogen peroxide, 1000 GW: 1000 grain weight, SDW: shoot dry weight, RFW: root fresh weight, Chl a: chlorophyll a, T Chl: total chlorophyll, Chl b: chlorophyll b, RDW: root dry weight, SL: shoot length, SFW: shoot fresh weight, S K: shoot potassium, RL: root length, S Ca: shoot calcium).

To reveal total variability, PCA analysis was performed and different variability dimensions were estimated. Among these, first two (Dim1 and Dim2) encompassed about 85.3% of the total variability explained by different morphological, agronomic and biochemical parameters. To comprehend the relationship among these variables under Proline and Nickel treatment, position of each variable was shown in PCA biplot based on both individual and variable projections (Fig. 7). Based on individual projections, factor analysis was performed in 2 × 2 factorials arrangements and was plotted for Nickel (Ni1 and Ni2) and Proline (Pr1 and Pr2) treatments. This led to four treatment combinations i.e., Ni1Pr1, Ni1Pr2, Ni2Pr2 and Ni2Pr1 which were distributed in II, I, III and IV quadrants respectively. To further comprehend the relationship with plant characteristics, these were plotted as vectors and degree of correlation as measure of angle in PCA biplot (Fig. 7). Our results showed that a group of plant variables including S Ca, RL, RDW, Chl a, Chl b, SFW, SL, SK, SDW and RFW were distributed in first quadrant showing positive correlation to Dim1 (67.5%) while biochemical parameters like Flavonoids, SOD, POD, CAT, AsA, TSS and TSP were positively correlated to Dim2 (17.8%). On contrary, variables like S Na, MDA and H_2_O_2_ were negatively related to both Dim1 and Dim2 (Fig. 7). However, no clear pattern was observed for morphological, agronomic or biochemical parameters in PCA biplot.

**Figure 8:**
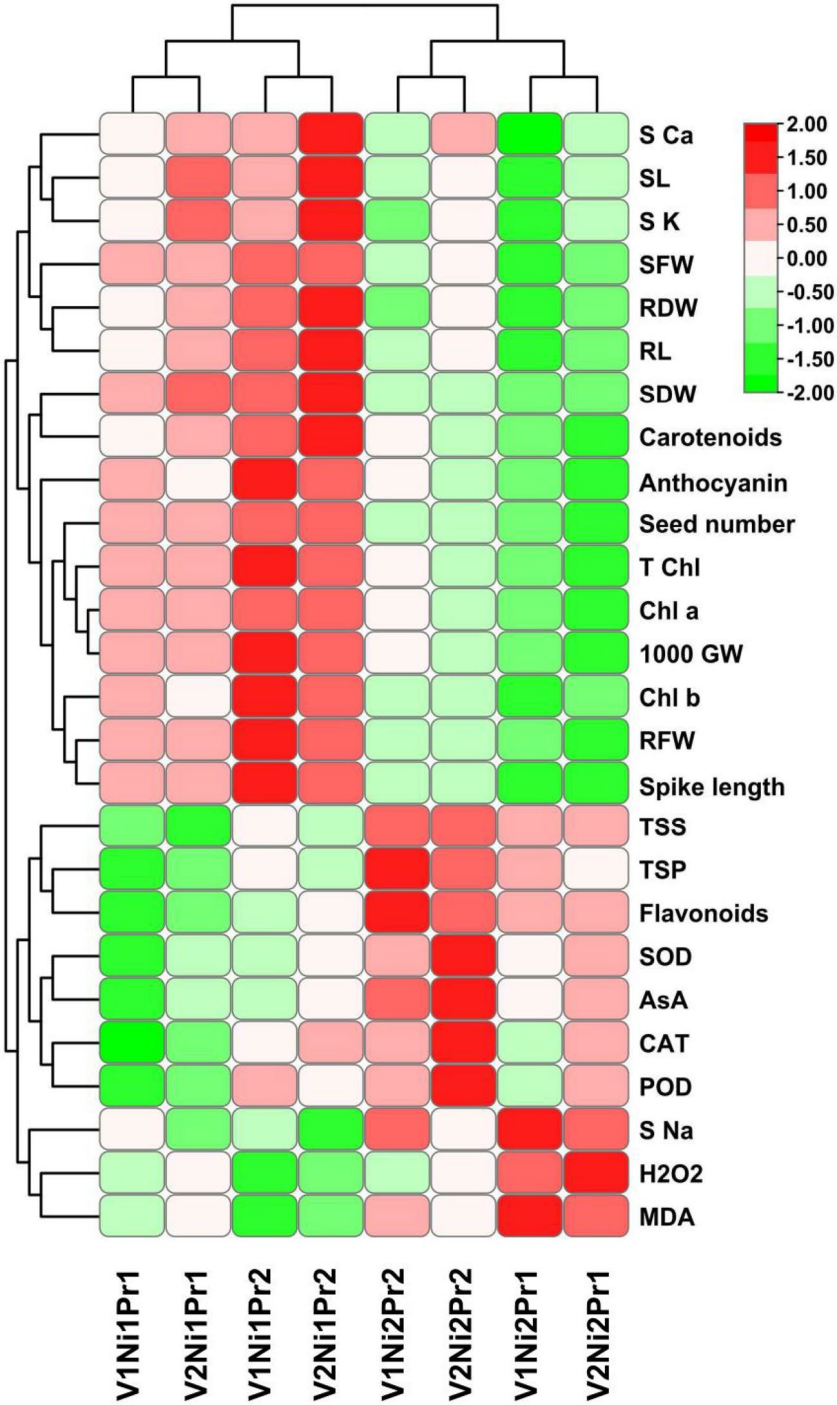
Heat plot showing mean performance of various plant characteristics under Proline and Nickel treatments. (CAT: catalase, POD: peroxidase, AsA: Ascorbic acid, SOD: superoxide dismutase, TSP: total soluble proteins, TSS: total soluble sugars, S Na: shoot sodium, MDA: malondialdehyde, H_2_O_2_: hydrogen peroxide, 1000 GW: 1000 grain weight, SDW: shoot dry weight, RFW: root fresh weight, Chl a: chlorophyll a, T Chl: total chlorophyll, Chl b: chlorophyll b, RDW: root dry weight, SL: shoot length, SFW: shoot fresh weight, S K: shoot potassium, RL: root length, S Ca: shoot calcium).

To compare mean performance under different treatments (Variety, Nickel and Proline levels) in eight different treatment combinations (V1Ni1Pr1, V1Ni1Pr2, V1Ni2Pr1, V1Ni2Pr2, V2Ni1Pr1, V2Ni1Pr2, V2Ni2Pr1, V2Ni2Pr2) for all studied variable and log normalized data was plotted as heat map (Fig. 8). The results showed that based on mean performance of plant variables, eight treatment combinations could be divided into two distinct categories. All treatment combinations with Nickel level 1 (Ni1) like V1Ni1Pr1, V1Ni1Pr2, V2Ni1Pr1 and V2Ni1Pr2 were placed in one group which demonstrated higher mean performance for S Ca, SL, S K, SFW, RDW, RL, SDW, RFW, Seed number, 1000 GW, Spike length and few biochemical parameters like carotenoids, Anthocyanin, T Chl, Chl a and Chl b (Fig. 8). Similarly, all treatment combinations with Nickel level 2 (Ni2) including V1Ni2Pr1, V1Ni2Pr2, V2Ni2Pr1 and V2Ni2Pr2 were placed in same group which depicted higher mean values for majority of biochemical parameters like TSS, TSP, Flavonoids, SOD, AsA, CAT, POD, H_2_O_2_ and MDA while all other variables showed lower mean values (Fig. 8).

**Figure 9:**
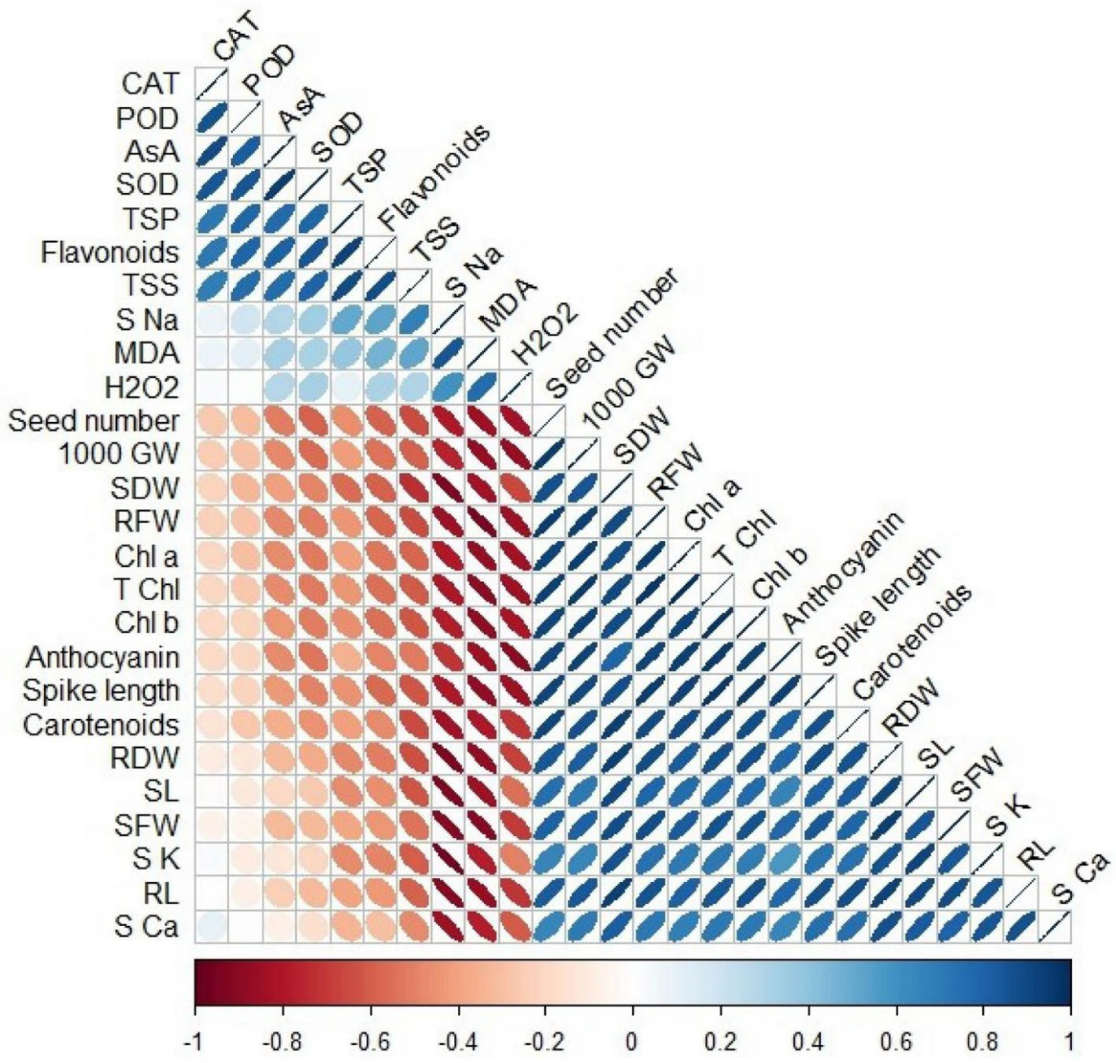
Correlation plot showing correlation matrix for various variables under Nickel and Proline treatments. Correlogram based on Pearson correlation. Only significant correlations (*p* < 0.05) are colored. Color gradient shows degree of relationship while positive and negative correlations were presented with different colors. (CAT: catalase, POD: peroxidase, AsA: Ascorbic acid, SOD: superoxide dismutase, TSP: total soluble proteins, TSS: total soluble sugars, S Na: shoot sodium, MDA: malondialdehyde, H_2_O_2_: hydrogen peroxide, 1000 GW: 1000 grain weight, SDW: shoot dry weight, RFW: root fresh weight, Chl a: chlorophyll a, T Chl: total chlorophyll, Chl b: chlorophyll b, RDW: root dry weight, SL: shoot length, SFW: shoot fresh weight, S K: shoot potassium, RL: root length, S Ca: shoot calcium).

The correlation analysis identified three main groups’ correlations among the different variables. The first group includes higher positive and significant correlations between biochemical parameters like CAT, POD, AsA, SOD, TSP, Flavonoids and TSS along with lower positive non-significant correlation values for MDA and S Na and H_2_O_2_. The second larger group showed significant negative correlation values among Seed Number, 1000 GW, SDW, RFW, T Chl, Chl a, Chl b, Anthocyanins, Spike length, Carotenoids, RDW, SL SFW, S K, RL and S Ca (Fig. 9).

## Discussion

### Plant growth and biomass

The risk for plant growth and development increased as a result of the unrestrained anthropogenic activities that added HMs to agricultural soils^51^. There have been reports on the negative effects of Ni at higher concentrations on the plant life cycle and its necessity for plants at lower concentrations^52–54^. Nevertheless, little research has been done on the negative effects of Ni toxicity and how plants can adapt to it. Growing wheat and other significant cereal crops in Ni-contaminated soil might pose substantial risks to the health of people, animals, and plants. Although Ni is a so-called vital microelement, too much of it can be hazardous^55^. Plant growth and photosynthetic capacity are also decreased as a result of Ni toxicity. In addition, it causes oxidative stress, impedes the intake of other metals, and suppresses the metabolism of nitrogen as well as enzymatic and mitotic processes. *Arabidopsis thaliana* (L.) was affected by a high Ni^2+^ concentration in the growth medium, which caused geotropism deficiencies by inhibiting root cell elongation^56^. According to Wang and Zhou^57^, plant tolerance to heavy metals is frequently evaluated based on the degree of root or shoot growth restriction produced by the existence of undesirable metal/s in the growing media. The current findings also support the previous findings by exhibiting reduced biomass attributes wheat plants under Ni^2+^ stress, as demonstrated by many scientists previously^37,58^. Nickel treated plants with a toxic concentration showed deprived root growth because of reticence of mitotic activity, thus restrict the overall growth of the plants^59^. Ni distribution in plant tissues is an indication of a change in chemical form resulting from complexation with plant-produced ligands. Studies on the uptake of HMs by plants have shown that HMs can also be transported passively from roots to shoots through the xylem vessels^60^. Nickel was also likely to have adverse effects on various metabolic and biochemical processes, as well as on plant cell proliferation, mitotic cell division, and cell wall flexibility^61,62^. These molecular disbalance resulted in arrested shoot and root weight as well as growth. To improve plant's adaptability against Ni^2+^ stress, certain signal molecules could be applied exogenously to serve as a potent solution. Among many signal molecules, proline is a notable biological signal molecule in plant tissues to regulate numerous growth and development processes. In stressed plants, increased proline levels in the roots and shoots may lead to osmoregulation. Proline is essential for maintaining membrane integrity, controlling cytosolic pH, regulating osmosis, stabilizing enzymes, and scavenging free radicals^63^. Categorically, Pro performs three important functions under stress in addition to being a great osmolyte: it chelates metals, works as potential antioxidant, and signaling molecule. The stimulation of growth in response to exogenous proline treatment could be attributed to changes in net photosynthetic rate and gaseous exchange characteristics, which improves the osmoregulatory mechanism of plant with and without stress^64,65^. The Pro has considerably defeated the Ni and salinity-induced toxic effects at early growing stage, water status, and photosynthetic attributes. The Pro is efficient in regulating polyamine metabolism to create tolerance in pea against Ni^66^.

The toxic concentration of Ni caused a drastic reduction of dry biomass and yield occurred was found to be different for different plant species. The current study also demonstrated the significant reduction of yield, and yield-related components under Ni^2+^ stress; while the exogenous application of Pro statistically stimulated the yield attributes, when compared with control. Previously, it has been documented that Ni^2+^ toxicity imparts negative consequences on mung bean yield due to phenomenon reduction of its morpho-physiological and biochemical attributes^67^.

### Lipid peroxidation and reactive oxygen species scavenging by proline under Ni stress

In this study, we found inhibition of gaseous, and photosynthetic attributes at 100 mg kg^−1^ concentration of Ni in wheat. Toxicity of Ni^2+^ most likely impair photosynthetic process by interfering with membrane selectivity, CO_2_ fixation, ultrastructure of chloroplast, and electron transport processes^68^. We recommended that higher transpiration rate indicated higher rate of translocation of Ni from roots to the shoot^69–71^. Proline mitigated the Ni^2+^ toxicity in wheat seedlings by improving photosynthetic attributes^72^.

Nickel convincingly influences metabolic processes by accumulating higher rate ROS, which induce oxidative stress in plants^73^. Variety of plants has displayed this phenomenon of increased production of ROS under abiotic stress. The higher concentration of proline inhibits pyroline-5-carboxylate synthetase and generate ROS causing growth inhibition in plants^69^. Some experiments recommended that 20 mM endogenous proline can completely switch off singlet oxygen^74^. Proline functions as a protein compatible osmolyte, scavenging free toxic oxygen and nitrogen radicals, buffering cellular redox potential to stabilize compartmental structures, membranes, and other inclusions. Our results recommended the positive role of Pro in wheat facing Ni^2+^ stress, might be attributed to upregulation of antioxidant enzymes against ROS in plants. Proline also has detrimental role in maintaining osmotic balance by keeping ionic homeostasis, successfully scavenging ROS, stabilizing antioxidant enzymes that minimize oxidative damage, and enhancing growth and yield^75^.

The higher accumulation of proline in the plant is a response to the induction of stress that can cause the plant to show tolerance mechanism. Proline accumulated in response to metals stress in many plants particularly against cadmium in chickpea, and olive, Ni in pea, and aluminium in trifoliate orange^76,77^. The application of Ni inhibited essential elements (N, P, K) and showed higher accumulation of MDA under Ni^2+^ stress^78^. The phytotoxicity induced by Ni on different growth and physiological parameters of wheat was mitigated by Pro as indicated by the higher values of the shoot and root growth and scavenge the plants from harmful ROS and limiting the lipid peroxidation.

### Micronutrient regulation by proline under Ni stress

Plants that are exposed to heavy metals excessively have lower concentrations of macro- and micronutrients. Plant growth and development require the correct levels of Ni and other minerals. Because Ni has the same ionic charge as Mg^2+^, Fe^2+^, and Zn^2+^, it exhibits properties comparable to those of these metals^79^. Nickel competes with both macronutrients (Ca, Mg) and micronutrients (Fe, Cu, and Zn) in plant sorption and transpiration because of their comparable properties^80^. Due to these similarities, a high Ni content prevents these nutrients from being absorbed, translocated, and sorption by plants^81^. Our findings support earlier research showing that Ni toxicity dramatically reduced the quantities of nutrients in wheat shoots. Proline has certainly improved the nutrient concentration both in stressed and non-stressed wheat seedlings. According to Zouari et al.^82^, exogenous proline also plays a protective role in osmotic adjustment maintenance, ionic homeostasis preservation, efficient ROS scavenging, antioxidant enzyme stabilization that reduces oxidative damage, plant photosynthetic rate enhancement, and growth and yield enhancement.

## Conclusion

Our objective regarding current study was to examine the hypothesis that Pro can mitigate the deleterious effect of Ni toxicity in wheat seedling. Our findings show that Pro can upregulate both morphological and biochemical parameters in such a way that enhances the plant tolerance to Ni pollution. This regulatory mechanism was clearly embodied in improving the chlorophyll pigments that was associated with the concomitant increase in the plant biomass, higher ionic uptake, and yield of both wheat cultivars with and without Ni stress. This alleviation effects not only enhanced photosynthesis and biomass, but also regulated the biosynthesis and accumulation of flavonoids, total soluble sugars, and ascorbic acid. Overall, this study offers a significant insight upon the potent ability of Pro to manipulate osmoregulatory metabolism to tolerate contamination with other heavy metal, particularly Ni for other significant cereal crops.

## Data Availability

All data generated or analyzed during this study are included in this published article.

## References

[1] Haider FU, Wang X, Zulfiqar U, Farooq M, Hussain S, Mehmood T, Naveed M, Li Y, Liqun C, Saeed Q, Ahmad I (2022). Biochar application for remediation of organic toxic pollutants in contaminated soils An update. Ecotoxicol. Environ. Safety.

[2] Zulfiqar U, Jiang W, Xiukang W, Hussain S, Ahmad M, Maqsood MF, Ali N, Ishfaq M, Kaleem M, Haider FU, Farooq N (2022). Cadmium phytotoxicity, tolerance, and advanced remediation approaches in agricultural soils; a comprehensive review. Front. Plant Sci..

[3] Zulfiqar U, Ayub A, Hussain S, Waraich EA, El-Esawi MA, Ishfaq M, Ahmad M, Ali N, Maqsood MF (2022). Cadmium toxicity in plants: Recent progress on morpho-physiological effects and remediation strategies. J. Soil Sci. Plant Nutr..

[4] Farhat F, Arfan M, Tariq A, Riaz R, Naila H (2021). Moringa leaf extract and ascorbic acid evoke potentially beneficial antioxidants especially phenolics in wheat grown under cadmium stress. Pak. J. Bot.

[5] Burakov AE, Galunin EV, Burakova IV, Kucherova AE, Agarwal S, Tkachev AG, Gupta VK (2018). Adsorption of heavy metals on conventional and nanostructured materials for wastewater treatment purposes: A review. Ecotoxicol. Environ. Safety.

[6] Zulfiqar U, Haider FU, Ahmad M, Hussain S, Maqsood MF, Ishfaq M, Shahzad B, Waqas MM, Ali B, Tayyab MN, Ahmad SA (2023). Chromium toxicity, speciation, and remediation strategies in soil-plant interface: A critical review. Front. Plant Sci..

[7] Zulfiqar U, Haider FU, Maqsood MF, Mohy-Ud-Din W, Shabaan M, Ahmad M, Kaleem M, Ishfaq M, Aslam Z, Shahzad B (2023). Recent advances in microbial-assisted remediation of cadmium-contaminated soil. Plants.

[8] Nagajyoti PC, Lee KD, Sreekanth TVM (2010). Heavy metals, occurrence and toxicity for plants: A review. Environ. Chem. Lett..

[9] Zulfiqar U, Farooq M, Hussain S, Maqsood M, Hussain M, Ishfaq M, Ahmad M, Anjum MZ (2019). Lead toxicity in plants: Impacts and remediation. J. Environ. Manag..

[10] Asati A, Pichhode M, Nikhil K (2016). Effect of heavy metals on plants: an overview. Int. J. Appl. or Innov. Eng. Manag..

[11] Rizwan M, Imtiaz M, Dai Z, Mehmood S, Adeel M, Liu J (2017). Nickel stressed responses of rice in Ni subcellular distribution, antioxidant production, and osmolyte accumulation. Environ. Sci. Pollut. Res..

[12] Fabiano CC, Tezotto T, Favarin JL, Polacco JC, Mazzafera P (2015). Essentiality of nickel in plants: a role in plant stresses. Front. Plant Sci..

[13] Aziz H, Sabir M, Ahmad HR, Aziz T, Zia-ur-Rehman M, Hakeem KR, Ozturk M (2015). Alleviating effect of calcium on nickel toxicity in rice. Clean Soil Air Water..

[14] Kumar A, Jigyasu DK, Subrahmanyam G, Mondal R, Shabnam AA, Cabral-Pinto MM, Malyan SK, Chaturvedi AK, Gupta DK, Fagodiya RK, Khan SA (2021). Nickel in terrestrial biota: comprehensive review on contamination, toxicity, tolerance and its remediation approaches. Chemosphere..

[15] Lock K, Van Eeckhout H, De Schamphelaere KA, Criel P, Janssen CR (2007). Development of a biotic ligand model (BLM) predicting nickel toxicity to barley (*Hordeum vulgare*). Chemosphere..

[16] Mustafa A, Zulfiqar U, Mumtaz MZ, Radziemska M, Haider FU, Holatko J, Hammershmiedt T, Naveed M, Ali H, Kintl A, Saeed Q (2023). Nickel (Ni) phytotoxicity and detoxification mechanisms: A review. Chemosphere..

[17] Fiala R, Fialova I, Vaculík M, Luxova M (2021). Effect of silicon on the young maize plants exposed to nickel stress. Plant Physiol. Biochem..

[18] Valivand M, Amooaghaie R, Ahadi A (2019). Seed priming with H2S and Ca2+ trigger signal memory that induces cross-adaptation against nickel stress in zucchini seedlings. Plant Physiol. Biochem..

[19] Rohman MM, Begum S, Talukder MZA, Akhi AH, Amiruzzaman M, Ahsan A (2016). Drought sensitive maize inbred shows more oxidative damage and higher ROS scavenging enzymes, but not glyoxalases than a tolerant one at seedling stage. Plant Omics.

[20] Raja V, Majeed U, Kang H, Andrabi KI, John R (2017). Abiotic stress: Interplay between ROS, hormones and MAPKs. Environ. Exp. Bot..

[21] Farhat F, Arfan M, Wang X, Tariq A, Kamran M, Tabassum HN, Tariq I, Mora-Poblete F, Iqbal R, El-Sabrout AM, Elansary HO (2022). The impact of bio-stimulants on Cd-stressed wheat (*Triticum aestivum* L.): Insights into growth, chlorophyll fluorescence, Cd accumulation, and osmolyte regulation. Front. Plant Sci..

[22] Farhat F, Ashaq N, Noman A, Aqeel M, Raja S, Naheed R, Maqsood MF, Haider I, Tariq A (2023). Exogenous application of Moringa leaf extract confers salinity tolerance in sunflower by concerted regulation of antioxidants and secondary metabolites. J. Soil Sci. Plant Nutr..

[23] Gupta R, Meghwal M, Prabhakar PK (2021). Bioactive compounds of pigmented wheat (*Triticum aestivum*): Potential benefits in human health. Trends Food Sci. Technol..

[24] Fao, IFAD, Unicef, WFP, and WHO. The state of food security and nutrition in the world 2019. Safeguarding against economic slowdowns and downturns. Rome: FAO (2019)

[25] Katileviciute A, Plakys G, Budreviciute A, Onder K, Damiati S, Kodzius R (2019). A sight to wheat bran: High value-added products. Biomolecules.

[26] Shewry PR, Hey SJ (2015). The contribution of wheat to human diet and health. Food Energy Secur..

[27] Taghouti M, Gaboun F, Nsarellah N, Rhrib R, El-Haila M, Kamar M (2010). Genotype x environment interaction for quality traits in durum wheat cultivars adapted to different environments. Afr. J. Biotechnol..

[28] Rozbicki J, Ceglińska A, Gozdowski D, Jakubczak M, Cacak-Pietrzak G, Mądry W (2015). Influence of the cultivar, environment and management on the grain yield and bread-making quality in winter wheat. J. Cereal. Sci..

[29] Wang Y, Wang S, Nan Z, Ma J, Zang F, Chen Y (2015). Effects of Ni stress on the uptake and translocation of Ni and other mineral nutrition elements in mature wheat grown in Sierozems from northwest of China. Environ. Sci. Pollut. Res..

[30] Nazia K, Khalid N, Khalid H, Bhatti KH, Siddiqi EH, Aqsa T (2014). Effect of exogenous applications of glycine betaine on growth and gaseous exchange attributes of two maize (*Zea mays* L.) cultivars under saline conditions. World Appl. Sci. J..

[31] Yildiztugay E, Ozfidan-Konakci C, Kucukoduk M, Duran Y (2014). Variations in osmotic adjustment and water relations of sphaerophysakotschyana: Glycine betaine, proline and choline accumulation in response to salinity. Bot. Stud..

[32] Noreen S, Faiz S, Akhter MS, Shah KH (2019). Influence of foliar application of osmoprotectants to ameliorate salt stress in sunflower (*Helianthus annuus* L.). Sarhad. J. Agric..

[33] Sofy MR, Seleiman MF, Alhammad BA, Alharbi BM, Mohamed HI (2020). Minimizing adverse effects of pb on maize plants by combined treatment with jasmonic, salicylic acids and proline. Agronomy.

[34] Hayat S, Hayat Q, Alyemeni MN, Wani AS, Pichtel J, Ahmad A (2012). Role of proline under changing environments: A review. Plant Signal. Behav..

[35] Rejeb KB, Abdelly C, Savouré A (2014). How reactive oxygen species and proline face stress together. Plant Physiol. Biochem..

[36] Bandehagh A, Valizadeh M, Ghaffari M, Jahangir F, Dehganian Z (2019). Pattern of antioxidant enzyme activities under drought stress and exogenous application of proline in sunflower. J. Crop Prod..

[37] Shahid S, Shahbaz M, Maqsood MF, Farhat F, Zulfiqar U, Javed T, Fraz Ali M, Alhomrani M, Alamri AS (2022). Proline-induced modifications in morpho-physiological, biochemical and yield attributes of pea (*Pisum sativum* L.) cultivars under salt stress. Sustainability..

[38] Abdelaal KA, Attia KA, Alamery SF, El-Afry MM, Ghazy AI, Tantawy DS, Al-Doss AA, El-Shawy ESE, Abu-Elsaoud M, A. and Hafez, Y.M. (2020). Exogenous application of proline and salicylic acid can mitigate the injurious impacts of drought stress on barley plants associated with physiological and histological characters. Sustainability.

[39] AlKahtani MD, Hafez YM, Attia K, Rashwan E, Husnain LA, AlGwaiz HI, Abdelaal KA (2021). Evaluation of silicon and proline application on the oxidative machinery in drought-stressed sugar beet. Antioxidants.

[40] Meier U (2001). Growth stages of mono-and dicotyledonous plants.

[41] Arnon DI (1949). Copper enzyme in isolated chloroplasts. Polyphenol oxidase in *Beta vulgaris*. Plant Physiol..

[42] Chance, B. and Maehly, A.C. Assay of catalases and peroxidases*.* 764–775. (1955) 10.1002/9780470110171.ch14 13193536

[43] Spitz DR, Oberly LW (2001). Measurement of MnSOD and CuZnSOD activity in mammalian tissue homogenates. Curr. Protoc. Toxicol..

[44] Bradford MM (1976). A rapid and sensitive method for the quantitation of microgram quantities of protein utilizing the principle of protein-dye binding. Anal. Biochem..

[45] Velikova V, Yordanovand I, Edreva A (2000). Oxidative stress and some antioxidant systems in acid rain-treated bean plants: protective roles of exogenous polyamines. Plant Sci..

[46] Cakmak I, Horst JH (1991). Effects of aluminum on lipid peroxidation, superoxide dismutase, catalase and peroxidase activities in root tips of soybean (*Glycine max*). Physiol. Plant..

[47] Stark, D. and V. Wray. Anthocyanins. In: Methods in plant biology, Plant Phenolics, I, pp. 32–356. J. B. Harborne (Ed.). Acdemic Press/Harcourt Brace Jovanovich, London. (1989)

[48] Yoshida S, Forno D, Cock J, Gomez K (1976). Determination of sugars and starch in plant tissue. Laboratory manual for physiological studies of rice. Int. Rice Res. Inst..

[49] Zhishen J, Mengcheng T, Jianming W (1999). The determination of flavonoid contents in mulberry and their scavenging effects on superoxide radicals. Food Chem..

[50] Mukherjee SP, Choudhuri MA (1983). Implications of water stress induced changes in the levels of endogenous ascorbic acid and hydrogen peroxide in Vigna seedlings. Physiol. Plant.

[51] Kn K, Ga GA, Alam P, Azzam MA, Balawi TA, Raja V (2022). Mitigation of negative effects of chromium (VI) toxicity in Faba bean (*Vicia faba*) plants through the supplementation of Kinetin (KN) and gibberellic Acid (GA3). Plants..

[52] Khan, M. I. R., & Khan, N. A. (Eds.). Reactive oxygen species and antioxidant systems in plants: role and regulation under abiotic stress. Singapore: Springer Singapore. (2017)

[53] Ghori NH, Ghori T, Hayat MQ, Imadi SR, Gul A, Altay V, Ozturk M (2019). Heavy metal stress and responses in plants. Int. J. Environ. Sci. Technol..

[54] Georgiadou EC, Kowalska E, Patla K, Kulbat K, Smolińska B, Leszczyńska J, Fotopoulos V (2018). Influence of heavy metals (Ni, Cu, and Zn) on nitro-oxidative stress responses, proteome regulation and allergen production in basil (*Ocimum basilicum* L.) plants. Front. Plant Sci..

[55] Farhat F, Fry SC (2023). Copper, cadmium and nickel pollution inhibit growth and promote ascorbate catabolism in cell cultures of Arabidopsis thaliana and *Zea mays*. Plant Biosyst. Int. J. Dealing Aspects Plant Biol..

[56] Lešková A, Zvarík M, Araya T, Giehl RFH (2020). Nickel toxicity targets cell wall-related processes and PIN2-mediated auxin transport to inhibit root elongation and gravitropic responses in Arabidopsis. Plant Cell Physiol..

[57] Rathor G, Chopra N, Adhikari T (2014). Effect of variation in nickel concentration on growth of maize plant: A comparative over view for pot and hoagland culture. Res. J. Chem. Sci. ISSN.

[58] Wang M, Zhou Q (2005). Single and joint toxicity of chlorimuron-ethyl, cadmium, and copper acting on wheat *Triticum aestivum*. Ecotoxicol. Environ. Saf..

[59] Siddiqui MH, Al-Whaibi MH, Basalah MO (2011). Interactive effect of calcium and gibberellin on nickel tolerance in relation to antioxidant systems in *Triticum aestivum* L. Protoplasma.

[60] Gajewska E, Skłodowska M, Słaba M, Mazur J (2006). Effect of nickel on antioxidative enzyme activities, proline and chlorophyll contents in wheat shoots. Biol. Plant..

[61] Gajewska E, Skłodowska M (2008). Differential biochemical responses of wheat shoots and roots to nickel stress: antioxidative reactions and proline accumulation. Plant Growth Regul..

[62] Yang J, Wang M, Jia Y, Gou M, Zeyer J (2017). Toxicity of vanadium in soil on soybean at different growth stages. Environ. Pollut..

[63] Islam MM, Hoque MA, Okuma E, Banu MNA, Shimoishi Y, Nakamura Y (2009). Exogenous proline and glycinebetaine increase antioxidant enzyme activities and confer tolerance to cadmium stress in cultured tobacco cells. J. Plant Physiol..

[64] Agami RA, Medani RA, Abd El-Mola IA, Taha RS (2016). Exogenous application with plant growth promoting rhizobacteria (PGPR) or proline induces stress tolerance in basil plants (*Ocimum basilicum* L.) exposed to water stress. Int. J. Environ. Agri. Res..

[65] Uruç Parlak K (2016). Effect of nickel on growth and biochemical characteristics of wheat (*Triticum aestivum* L.) seedlings. NJAS Wageningen J. Life Sci..

[66] Pushpam AK, Mini M, Amutha S (2020). Assessing biochemical changes in exogenous application of osmoprotectants in amelioration of water stress in black gram (*Vigna mungo* L.). Int. J. Chem. Stud..

[67] Shahzad K, Ali A, Ghani A, Nadeem M, Khalid T, Nawaz S (2023). Exogenous application of proline and glycine betaine mitigates nickel toxicity in mung bean plants by up-regulating growth, physiological and yield attributes. Pak. J. Bot..

[68] Kumar S, Wang M, Liu Y, Fahad S, Qayyum A, Jadoon SA (2022). Nickel toxicity alters growth patterns and induces oxidative stress response in sweetpotato. Front. Plant Sci..

[69] Idrees M, Naeem M, Khan MN, Aftab T, Khan MM, Moinuddin (2012). Alleviation of salt stress in lemongrass by salicylic acid. Protoplasma..

[70] Yang J-Y, Tang Y (2015). Accumulation and biotransformation of vanadium in opuntia microdasys. Bull. Environ. Contam. Toxicol..

[71] Abedini M, Mohammadian F (2018). Vanadium effects on phenolic content and photosynthetic pigments of sunflower. South-Western J. Hortic. Biol. Env..

[72] Aqeel M, Khalid N, Tufail A, Ahmad RZ, Akhter MS, Luqman M (2021). Elucidating the distinct interactive impact of cadmium and nickel on growth, photosynthesis, metal-homeostasis, and yield responses of mung bean (*Vigna radiata* L.) varieties. Environ. Sci. Pollut. Res..

[73] Sreekanth TVM, Nagajyothi PC, Lee KD, Prasad T (2013). Occurrence, physiological responses and toxicity of nickel in plants. Int. J. Environ. Sci. Technol..

[74] Miller G, Honig A, Stein H, Suzuki N, Mittler R, Zilberstein A (2009). Unraveling Δ1-pyrroline-5-carboxylate-proline cycle in plants by uncoupled expression of proline oxidation enzymes. J. Biol. Chem..

[75] Alia MP, Matysik J (2001). Effect of proline on the production of singlet oxygen. Amino Acids..

[76] Yan L, Riaz M, Jiang C (2020). Exogenous application of proline alleviates B-deficiency-induced injury while aggravates aluminum toxicity in trifoliate orange seedlings. Sci. Hortic..

[77] Siddiqui MH, Al-Whaibi MH, Ali HM, Sakran AM, Basalah MO, AlKhaishany MYY (2013). Mitigation of nickel stress by the exogenous application of salicylic acid and nitric oxide in wheat. Aust. J. Crop Sci..

[78] Ghosh UK, Islam MN, Siddiqui MN, Cao X, Khan MAR (2022). Proline, a multifaceted signalling molecule in plant responses to abiotic stress: understanding the physiological mechanisms. Plant Biol..

[79] Kazemi N, Khavari-Nejad RA, Fahimi H, Saadatmand S, Nejad-Sattari T (2010). Effects of exogenous salicylic acid and nitric oxide on lipid peroxidation and antioxidant enzyme activities in leaves of Brassica napus L under nickel stress. Sci. Hortic..

[80] Gajewska E, Bernat P, Długoński J, Skłodowska M (2012). Effect of nickel on membrane integrity, lipid peroxidation and fatty acid composition in wheat seedlings. J. Agron. Crop Sci..

[81] Ain Q, Akhtar J, Amjad M, Haq MA, Saqib ZA (2016). Effect of enhanced nickel levels on wheat plant growth and physiology under salt stress. Commun. Soil Sci. Plant Anal..

[82] Zouari, M., A.B. Hassena, L. Trabelsi, B.B. Rouina, R. Decou and P. Labrousse. Exogenous proline-mediated abiotic stress tolerance in plants: Possible Mechanisms. In: (Eds.): Hossain, M.A., V. Kumar, D.J. Burritt, M. Fujita and P.S.A. Mäkelä. Osmoprotectant-Mediated Abiotic Stress Tolerance in Plants. Springer, Cham, pp. 99–121. (2019)

