# Author Correction: Proline-mediated redox regulation in wheat for mitigating nickel-induced stress and soil decontamination

**DOI:** 10.1038/s41598-024-52695-z

**Published:** 2024-01-25

**Authors:** Nimra Atta, Muhammad Shahbaz, Fozia Farhat, Muhammad Faisal Maqsood, Usman Zulfiqar, Nargis Naz, Muhammad Mahmood Ahmed, Naveed Ul Hassan, Nazoora Mujahid, Abd El-Zaher M. A. Mustafa, Mohamed S. Elshikh, Talha Chaudhary

**Affiliations:** 1 Department of Botany, University of Agriculture, Faisalabad, Pakistan; 2 Department of Botany, Government College Women University, Faisalabad, Pakistan; 3 Department of Botany, The Islamia University of Bahawalpur, Bahawalpur, 63100 Pakistan; 4 Department of Agronomy, Faculty of Agriculture and Environment, The Islamia University of Bahawalpur, Bahawalpur, 63100 Pakistan; 5 Department of Bioinformatics, The Islamia University of Bahawalpur, Bahawalpur, 63100 Pakistan; 6 Department of Botany and Microbiology, College of Science, King Saud University, P.O. 2455, Riyadh, 11451 Saudi Arabia; 7 Faculty of Agricultural and Environmental Sciences, Hungarian University of Agriculture and Life Sciences, Godollo, 2100 Hungary

Correction to: *Scientific Reports* 10.1038/s41598-023-50576-5, published online 03 January 2024

In the original version of this Article Abd El‑Zaher M. A. Mustafa and Mohamed S. Elshikh were incorrectly affiliated with ‘Faculty of Agricultural and Environmental Sciences, Hungarian University of Agriculture and Life Sciences, Godollo 2100, Hungary’. The correct affiliation is listed below.

Department of Botany and Microbiology, College of Science, King Saud University, P.O. 2455, Riyadh 11451, Saudi Arabia.

In addition, Talha Chaudhary was incorrectly affiliated with ‘Department of Botany and Microbiology, College of Science, King Saud University, P.O. 2455, Riyadh 11451, Saudi Arabia’.

The correct affiliation is listed below.

Faculty of Agricultural and Environmental Sciences, Hungarian University of Agriculture and Life Sciences, Godollo 2100, Hungary

The original Article has been corrected.

